# A National Study Exploring the Association between Fasting Duration and Mortality among the Elderly

**DOI:** 10.3390/nu16132018

**Published:** 2024-06-26

**Authors:** Zhixuan Zhang, Hang Zhao, Zhengyu Tao, Meng Jiang, Jun Pu

**Affiliations:** Division of Cardiology, State Key Laboratory of Systems Medicine for Cancer, Renji Hospital, School of Medicine, Shanghai Jiao Tong University, 160 Pujian Road, Shanghai 200127, China; 19821838993@163.com (Z.Z.); hang_zhao2009@163.com (H.Z.); taozhengyu1998@126.com (Z.T.)

**Keywords:** time-restricted eating, fasting duration, mortality, elderly population, NHANES

## Abstract

(1) Background: The benefits of weight management are widely recognized, and prolonged fasting duration has become a common method for weight control. The suitability of time-restricted eating (TRE) for elderly individuals remains controversial. This study aims to examine the correlation between fasting duration and mortality within a nationally representative cohort of elderly individuals in the United States. (2) Methods: Data were extracted from a prospective cohort study conducted as part of the National Health and Nutrition Examination Survey (NHANES) from 2005 to 2018. Participants aged over 60 with complete data on dietary intake and mortality follow-up information were included. Fasting duration was assessed using two 24 h dietary recalls. All the participants were categorized into fasting duration quartiles. Mortality outcomes were ascertained through the National Death Index. Cox proportional hazards regression models were utilized to analyze the association between fasting duration and mortality. (3) Results: The final analysis included 10,561 elderly participants (mean age 69.89, 45.58% male). Individuals with the longest fasting duration (over 12.38 h) had a significantly higher risk of CVD mortality compared to those with a normal fasting duration (10.58–12.38 h). This elevated CVD mortality risk was particularly pronounced in males, individuals over 70 years old, and non-shift workers. A non-linear relationship was observed between fasting duration and all-cause mortality and CVD mortality. (4) Conclusions: Prolonged fasting periods are associated with a higher risk of CVD mortality in the elderly population, although this correlation is not evident for all-cause, cancer, or other-cause mortality. A fasting duration of 11.49 h correlates with the lowest mortality risk. Additionally, elderly individuals with the shortest fasting duration exhibit elevated hazard ratios for both cancer and other-cause mortality. As with any health intervention, clinicians should exercise caution when recommending a fasting regimen that is personalized to the health condition of people who are older. Further research through randomized controlled trials should be conducted to comprehensively investigate the impact of TRE on mortality.

## 1. Introduction

Obesity has evolved into a global epidemic over the past decade, impacting approximately 75% of individuals in the United States [1]. The current evidence suggests that obesity is intricately linked to the incidence of cardiovascular disease [2], diabetes [3], and other metabolic disorders [4]. With the growing awareness of the importance of weight management [5], it has become evident that even a slight reduction of 5% in one’s initial body weight can significantly mitigate, or even prevent, metabolic comorbidities among those who are overweight or obese [6].

Weight management strategies are multifaceted, ranging from dietary control and medication to lifestyle modifications [7]. Among dietary interventions, intermittent fasting (IF) has emerged as a promising approach. IF encompasses various methods that restrict the consumption of food to specific time windows each day or week, leading to extended periods of fasting [8]. Time-restricted eating (TRE), a subset of IF, involves confining daily eating to a set time window [9]. Common TRE protocols include the 16/8, 18/6, and 20/4 schedules, which allow, respectively, an 8 h, 6 h, and 4 h eating window, followed by a 16 h, 18 h, and 20 h fast [10]. These fasting intervals are not arbitrary; they are designed to align with circadian rhythms and metabolic cycles, which may potentially enhance weight loss and metabolic health [11]. Research indicates that a 6 h eating window (18 h of fasting) can lower blood pressure and fasting insulin levels, albeit with a trade-off of increased serum triglycerides [12]. Conversely, an 8 h eating window (16 h of fasting) has been shown to effectively reduce triglyceride levels [13]. The 16/8 eating schedule is particularly popular, mandating a daily fasting period of at least 16 h, with the remaining 8 h dedicated to eating without restrictions [14]. The choice of fasting interval can be tailored to individual lifestyles and preferences, with the potential to optimize metabolic health and weight management outcomes [15]. Nevertheless, it is essential to recognize that individual responses to these fasting intervals can vary, underscoring the need for further research to identify optimal fasting durations for different populations [16].

The application of TRE in the elderly population introduces a complex landscape of potential benefits and risks. While there is growing evidence that TRE can significantly aid in weight loss among the overweight elderly [17,18], its long-term effects on mortality and cardiometabolic health are not yet fully understood. One recent study indicated a potential correlation between prolonged fasting duration and an increased cardiovascular mortality risk in the general US population [19]. However, this finding may not be directly extrapolated to the elderly, as this demographic has not been the focus of such studies. Additionally, there is a need for caution regarding the potential for prolonged fasting to increase risks such as hypoglycemia in individuals with type 2 diabetes [20] and to contribute to malnutrition, reduced bone density, loss of lean mass, and constipation in the elderly [21,22,23]. These considerations highlight the complexity of applying TRE in the elderly population and the necessity for targeted research to fully understand its implications for this group.

The present study aimed to examine the association between fasting duration and mortality in a nationally representative cohort of elderly people in the United States. Using dietary data from the National Health and Nutrition Examination Survey (NHANES) from 2005 to 2018, we analyzed the long-term impact of fasting duration on all-cause and different cause-specific mortality in participants aged over 60 years old. A comprehensive understanding of the link between fasting duration and mortality will offer insights into the safety and feasibility of TRE in the elderly population.

## 2. Materials and Methods

### 2.1. Study Population

The NHANES is a large-scale, multistage, ongoing, nationally representative health survey of the civilian noninstitutionalized population in the United States [24]. Participants are invited to complete an interviewer-based questionnaire followed by a physical examination and laboratory measurements at a mobile examination center. The NHANES was approved by the National Center for Health Statistics (NCHS) Ethics Review Board. Written informed consent was obtained from all participants.

In this study, we extracted data from 2005 to 2018, covering 7 survey cycles and involving a total of 70,191 individuals. In accordance with the Center for Disease Control and Prevention’s definition of elderly individuals [25], all the participants aged over 60 (*n* = 13,481) were initially chosen. Those with missing dietary intake (*n* = 2906) or with missing mortality data (*n* = 14) were excluded.

### 2.2. Exposure Assessment

To determine the micro- and macronutrient intake, the NHANES employed the US Department of Agriculture’s Food and Nutrient Database for Dietary Studies to process dietary information. The participants underwent two 24 h dietary recalls. The first recall consisted of an in-person interview, while the second recall was conducted over the phone, with a time gap of 3 to 10 days after the initial dietary interview, though not consistently on the same day of each week. In the current study, we included those participants who had successfully completed interviews on both days, utilizing the average fasting duration of these two days. During each recall session, the participants were instructed to report all meals consumed from midnight to 11:59 p.m.

The length of the overnight fasting period was calculated by subtracting the time between the first and last consumption of calorie-containing (or 5 kcal) food or beverages during each 24 h dietary recall day from 24 h. The calculation equation used was as follows: 24 − time of last calorie intake + time of first calorie intake [26]. To determine the fasting periods, the food consumption duration was converted into hours over a 24 h period.

Furthermore, to establish a coherent framework for dietary analysis, the fasting duration was measured from the first intake after waking until the last intake before sleep, accounting for any late-night eating. Within the NHANES protocol, “breakfast” was defined as the initial meal post-awakening, with any intake preceding breakfast considered to be the final consumption before bedtime. If there was any calorie consumption before breakfast, the participant was considered to have a late-night eating pattern. Notably, our study identified 2347 participants exhibiting late-night eating patterns on day 1, and 2215 on day 2. For this late-night eating pattern, the fasting duration calculation entailed subtracting the initial calorie intake time from the breakfast consumption time [26].

Additionally, a comparative analysis scrutinized fasting durations and nutrient intake derived from two dietary recalls (Table S1). To provide a visual representation, a histogram was generated to illustrate the distribution of fasting durations among the study participants (Figure S1).

### 2.3. Outcome Ascertainment

The primary outcome of our study was mortality from various causes, including all-cause mortality, cancer (codes C00–C97), cardiovascular disease (CVD, classified by codes I00–I09, I11, I13, I20–I25, I26–I51, and I60–I69), and other causes in compliance with ICD-10 (10th revision of the international statistical classification of diseases).

Mortality status and cause of death were ascertained by the NHANES linked National Death Index public-access files (https://www.cdc.gov/nchs/data-linkage/mortality-public.htm) through 31 December 2019. The linkage of the NHANES survey data with the NDI, approved by the NCHS Research Ethics Review Board, involved ensuring that all the survey participants had sufficient identifying data, such as SSN, names, birth date, and sex, to be eligible for mortality linkage. Eligibility was indicated by the variable ELIGSTAT, with values of 0 (ineligible) or 1 (eligible). The linkage process used both deterministic and probabilistic methods, with the latter employing the Fellegi–Sunter method to estimate match probabilities and select the most probable matches. The linkage algorithm, developed using SAS 9.4, aimed to produce high-quality matches with minimal error.

The duration of follow-up was calculated from the date of examination at the mobile examination center to the occurrence of the recorded death.

### 2.4. Covariate Assessment

#### 2.4.1. Demographic Variables

All the demographic variables were based on the NHANES official settings. The demographic factors included age categories (60–64, 65–69, 70–74, or ≥75), educational attainment (below 9th grade, 9th–11th grade, high school graduate, some college or associate degree, or college or higher), ethnicity (Mexican American, other Hispanic, non-Hispanic White, non-Hispanic Black, or other race), marital status (married, widowed, divorced, separated, never married, or cohabiting), family poverty income ratio (≤1, >1, ≤3, or >3), and gender (male or female). Participants with a family poverty income ratio below 1.00 were classified as residing below the poverty threshold, following guidelines set by the US Census Bureau [27].

#### 2.4.2. Other Relevant Variables

Most of the disease variables and the sleep duration were derived from self-reported diagnoses and objective laboratory measurements. Other variables, such as alcohol use [28], body mass index (BMI) [29], dietary inflammatory index (DII) [30], reporter status [31], and shift work [32], were informed by previous literature. Variables related to meal timing, including the first and last meal of the day, were based on the average reported times of these meals.

Diabetes was defined by self-reported prior diagnosis, hemoglobin A1c levels (≥6.5%), fasting plasma glucose levels (≥7 mmol/L), or current use of diabetes medications or insulin [33]. Hypertension was identified by calculating the average of three consecutive blood pressure readings during the physical examination in the NHANES database. Hypertension was diagnosed if the systolic blood pressure was ≥140 mmHg or diastolic blood pressure was ≥90 mmHg [34]. Cardiovascular disease (CVD) was defined as a self-reported diagnosis of congestive heart failure, coronary heart disease, angina/angina pectoris, or heart attack. Responding “Yes” to any of these queries indicated the presence of CVD. Depressive symptoms over the preceding two weeks were determined using the Patient Health Questionnaire [35]. A score above 5 indicated the presence of depressive symptoms, designating the variable “depression” as “Yes”. The “chronic kidney disease (CKD)” variable was based on self-reported diagnosis (No or Yes) or an estimated glomerular filtration rate of <60 (mL/min/m^2^) [36]. The “smoking” variable was categorized into three levels: “now”, “former”, or “never”. “Now” denoted current smokers who had consumed more than 100 cigarettes in their lifetime and currently smoked occasionally or daily. “Former” referred to individuals who had smoked more than 100 cigarettes in their lifetime but had ceased smoking. “Never” indicated those who had smoked fewer than 100 cigarettes in their lifetime and were not current smokers. Alcohol use was categorized as follows: “mild drinker (≤1 drink per day for women or ≤2 drinks per day for men on average over the past year)”, “moderate drinker (≤2 drink per day for women or ≤3 drinks per day for men on average over the past year)”, “former drinker (had ≥12 drinks in 1 year and did not drink last year, or did not drink last year but drank ≥12 drinks in lifetime)”, “heavy drinker (>1 drink per day for women or >2 drinks per day for men on average over the past year)” and “never drinker (had <12 drinks in lifetime)” [28]. The body mass index (BMI) was calculated to evaluate the weight status of the participants. A BMI ≥ 25 kg/m^2^ indicated “overweight”, while a BMI of ≥30 kg/m^2^ indicated “obesity” [29]. The dietary inflammatory index (DII) was computed to evaluate the connection between the participants’ diets and health outcomes, spanning from blood levels of inflammatory cytokines to chronic illnesses [30]. “First meal” was categorized based on the average time of initial caloric intake of the day (7:37 a.m.), with 8 a.m. chosen as the threshold. “Late eating” was categorized based on the average time of final caloric intake of the day (8:15 p.m.), with 9 p.m. chosen as the threshold. We employed the expected ratio of energy intake (EI) to estimate the energy requirement (EER) and to delineate the “reporter status”, which was classified as “under-reporters”, “acceptable reporters”, or “over-reporters” of energy intake. The EER was computed using equations derived from the US Dietary Reference Intakes, assuming a “low active” level of physical activity. “Under-reporters”, “acceptable reporters”, and “over-reporters” were categorized based on their EI:EER ratios, with values less than 0.65 indicating under-reporting, values between 0.65 and 1.53 signifying acceptable reporting, and values exceeding 1.53 denoting over-reporting. This classification was applied to 2-day dietary recall data from the US adult population [31]. In addition, we established the categorization of individuals as “shift work”, a designation contingent upon responses to the query titled “which of the following best describes the hours you usually work at your main job or business?” within the Occupational Questionnaire Section. Specifically, the participants reporting engagement in evening/night shifts or partaking in rotating shift schedules were classified within the “yes” group, while those adhering to a consistent daytime regimen were classified as members of the “no” group [32]. The variable “sleep hour” was determined by the self-reported item: “How much sleep do you usually get at night?”. We also defined a variable called “breakfast skipping”, which referred to participants skipping breakfast for both days.

### 2.5. Statistical Analysis

Based on the NCHS’s recommendation, the dietary sampling weights were used in all the analyses to interpret the complex NHANES survey design. The survey-weighted test was applied to all analyses. Continuous variables were represented by the mean (standard error), and categorical variables were expressed as the weighted percentage. Demographic and clinical characteristics were compared among the groups with different categories of fasting duration using the Student’s *t*-test, analysis of variance with post hoc Bonferroni correction for continuous variables, the Mann–Whitney U test and Kruskal–Wallis test, or the chi-squared test for categorical variables, as appropriate.

The associations between fasting duration and all-cause and cause-specific mortality were investigated using multivariate Cox proportional hazards regression models. In selecting covariates, we employed the bidirectional stepwise regression method to determine the most fitting-adjusted variables for each model. Hazard ratios (HRs) and their corresponding 95% confidence intervals (CIs) were used to present the outcomes of the regression models. We implemented false discovery rate (FDR) correction for the correction of the *p* value in our main analyses to address the problem of multiple comparisons. Restricted cubic spline analyses were conducted to explore the potential non-linear associations between the fasting duration and all-cause and cause-specific mortality. Additionally, sensitivity analyses were performed within the gender, age, acceptable reporters, and non-shift worker subgroups. Furthermore, adjusted models were constructed to assess the link between CVD mortality and fasting duration, stratified by diabetes, BMI status, smoking habits, and the timing of the first meal and last meal. The results of these stratified analyses were visualized in forest plots.

Statistical analyses were carried out using R software 4.3 (http://www.R-project.org, The R Foundation, accessed on 25 March 2024). A two-sided *p* value less than 0.05 was statistically significant.

## 3. Results

### 3.1. Description of Study Participants

From 2005 to 2018, 13,481 consecutive participants aged over 60 from the NHANES datasets were extracted. The participants with incomplete dietary data (*n* = 2906) and mortality data (*n* = 14) were excluded. A total of 10,561 individuals were included in our final analysis. The fasting duration observed in the two-day dietary recall showed minimal disparity (Tables S1 and S2), with the majority of participants exhibiting a fasting duration of approximately 10 h (Figure S1).

Table 1 and Table S3 summarize the characteristics of the study cohort, stratified according to fasting duration quartiles. Detailed information can be found in Table S3. Compared to individuals with the shortest nighttime fasting duration (≤7.5 h, Quartile 1), those with the longest nighttime fasting duration (>12.38 h, Quartile 4) tended to be older, predominantly female, non-Hispanic Black, and widowed and tended to have a lower income-to-poverty level ratio and lower educational attainment. Individuals with longer fasting periods were also less prone to smoking or alcohol consumption and tended to start their first meal later as well as end their final meal earlier. The subgroup with the longest fasting duration was also correlated with a higher prevalence of diabetes, CKD, depression, CVD, hypertension, and higher dietary inflammation index values. Additionally, those with the longest fasting duration exhibited a lower EI:EER ratio, a lower proportion of acceptable reporters and shift workers, a longer sleeping duration, and a higher possibility of skipping breakfast.

### 3.2. Relationship between Fasting Duration and Mortality

**Figure 1:**
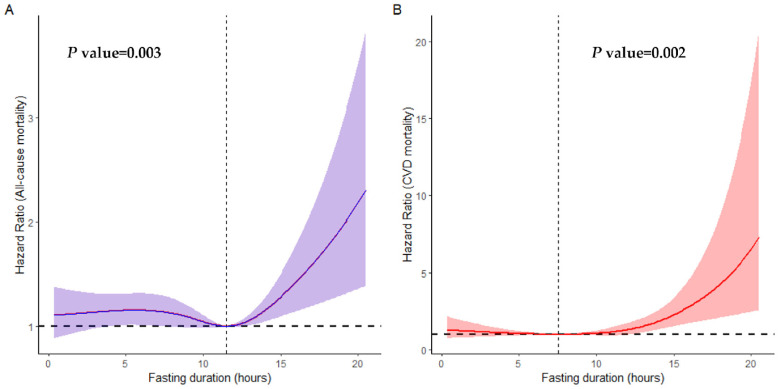
Non-linear association between fasting duration and all-cause (**A**) and CVD (**B**) mortality examined by multivariable Cox regression models. Solid red and purple lines represent hazard ratios adjusted for age (categorial), gender, educational level, marital status, ratio of income to poverty (categorial), ethnicity, diabetes, chronic kidney disease, body mass index, depression, smoking, use of alcohol, dietary inflammatory index, hypertension, cardiovascular disease, shift work, first meal, late eating, sleep hour, reporter status, and breakfast skipping, with light red and purple areas showing 95% confidence intervals derived from restricted cubic spline regressions with four knots. The dotted lines indicate the reference points set at zero, corresponding to 7.35 h for all-cause mortality and 11.49 h for CVD mortality.

We found that 2715 participants in the cohort passed away over a median follow-up period of 6.66 years. A non-linear association between fasting duration and all-cause as well as CVD mortality is depicted in Figure 1. We observed a statistically significant U-shaped relationship between fasting duration and all-cause (non-linear *p* value = 0.003), as well as CVD mortality (non-linear *p* value = 0.002). A fasting duration of 11.49 h was associated with the lowest all-cause mortality while that of 7.35 h was associated with the lowest CVD mortality rate. However, there was no association between fasting duration and cancer as well as other-cause mortality rates (non-linear *p* value > 0.05, Figure S2).

Additionally, we performed multivariable Cox regression in the younger population. After excluding participants with missing data on the fasting duration (*n* = 16,677) or mortality (*n* = 54) and those aged under 18 (*n* = 22,137) or over 60 (*n* = 10,561), there were 20,761 participants aged 18–59 from NHANES 2005–2018. We found that there was no significant association between the overnight fasting time and adjusted all-cause mortality in the younger participants (non-linear *p* value > 0.05, Figure S3).

Table 2 shows the correlations between the fasting duration quartiles and all-cause as well as cause-specific mortality. Quartile 3 was selected as the reference group for individuals not undergoing fasting, a decision informed by the observation that a fasting duration of 10 h demonstrated the highest frequency, as illustrated in Figure S1. After adjustment for age, gender, educational level, marital status, income-to-poverty ratio, ethnicity, diabetes, CKD, BMI, depression, smoking, alcohol use, dietary inflammation index, hypertension, CVD, shift work, first meal, late eating, sleep hour, reporter status, and breakfast skipping, Quartile 4 displayed significantly elevated CVD mortality (HR: 1.56, 95% CI: 1.08–2.25) compared to Quartile 3. Additionally, Quartile 1 was associated with an elevated hazard ratio for all-cause mortality (HR:1.41, 95% CI: 0.99–2.01) and other mortality (HR:1.44, 95% CI: 0.98–2.13), with *p* values just above the conventional threshold of 0.05. Examining the fasting duration as a continuous variable revealed that each additional hour of fasting duration correlated with a 5% elevation in cancer mortality (HR: 0.95, 95% CI: 0.91–0.98) and a 4% elevation in CVD mortality (HR: 1.04, 95% CI: 1.00–1.09).

### 3.3. Relationship between Fasting Duration and Mortality in Gender, Age, Acceptable Reporters, and Non-Shift Worker Subgroups

**Figure 2:**
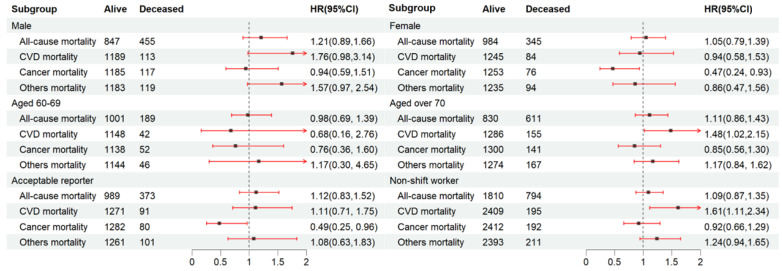
Subgroup analysis for the relationship between fasting duration and mortality among the elderly population. Values are n or hazard ratios (95% confidence intervals) of Quartile 4 (fasting duration > 12.38 h) with Quartile 3 (fasting duration > 10.58 h, ≤12.38 h) as the reference. Other mortality was adjusted for age (categorial), gender, educational level, marital status, ratio of income to poverty (categorial), ethnicity, diabetes, chronic kidney disease, body mass index, depression, smoking, alcohol use, dietary inflammatory index, shift work, first meal, late eating, sleep hour, reporter status, and breakfast skipping. CVD mortality and all-cause mortality were further adjusted (from other mortality) for hypertension and CVD. Cancer mortality was further adjusted (from other mortality) for cancer.

We further explored the link between the fasting duration and mortality risk among the gender, age, acceptable reporter, and non-shift worker subgroups. The participants with the longest fasting duration (Quartile 4) were compared to those who did not fast (Quartile 3) (Figure 2). We found that CVD mortality was substantially greater among individuals aged over 70 (HR: 1.48, 95% CI: 1.02–2.15) within Quartile 4 compared to Quartile 3. In contrast, a lower cancer mortality was noted in females (HR: 0.47, 95% CI: 0.24–0.93) and acceptable reporters (HR: 0.49, 95% CI: 0.25–0.96) within Quartile 4. The relationship between the fasting duration and CVD mortality remained constant among non-shift workers (HR: 1.61, 95% CI: 1.11–2.34).

### 3.4. Relationship between Fasting Duration and CVD Mortality Stratified by Diseases and Lifestyles

**Figure 3:**
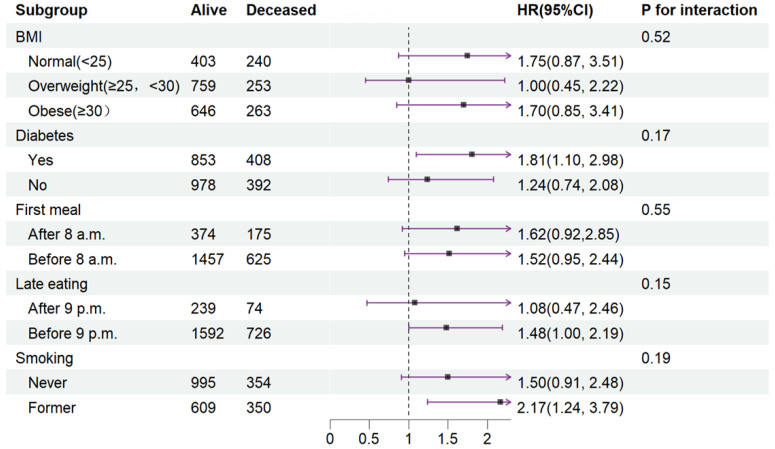
Stratified analysis of relationship between fasting duration and CVD mortality. Values are n or hazard ratios (95% confidence intervals) of Quartile 4 (fasting duration > 12.38 h) with Quartile 3 (fasting duration > 10.58 h, ≤12.38 h) as the reference. CVD mortality was adjusted for age (categorial), gender, educational level, marital status, ratio of income to poverty (categorial), ethnicity, chronic kidney disease, depression, alcohol use, dietary inflammatory index, shift work, sleep hour, reporter status, breakfast skipping, hypertension, and CVD. Abbreviation: CVD, cardiovascular disease.

To elucidate the influence of disease status or lifestyle on CVD mortality, Cox proportional regression models were conducted in Quartile 4 (with Quartile 3 as the reference) across various subgroups (Figure 3).

In terms of interaction effects, the impact of an extended fasting duration on CVD mortality was unaffected by BMI, diabetes, timing of the first meal and last meal, or smoking after full covariate adjustment (*P* interaction ranging from 0.17 to 0.55).

Utilizing nationally prospective cohort data, our study revealed a significant association between a prolonged night fasting duration and heightened CVD mortality, along with lower cancer mortality in the elderly population. Specifically, the CVD mortality risk was substantially prominent in non-shift workers and individuals aged over 70 with a fasting duration exceeding 12.38 h. Furthermore, we found that a fasting duration of 11.49 h was linked to the lowest overall mortality and 7.35 h to the lowest CVD mortality. To our knowledge, this is the first prospective analysis to explore the correlation between fasting duration and mortality among elderly adults, drawing from nationally representative U.S. population data. Our findings underscore the potential risks associated with TRE (time-restricted eating) for the elderly and imply that clinicians should exercise caution when advising TRE for those above 60 years old.

## 4. Discussion

Our study explored the relationship between fasting duration and mortality rates among elderly individuals in the United States. The key findings included the following: firstly, longer fasting durations (>12.38 h) were associated with a higher risk of cardiovascular disease (CVD) mortality, particularly among males, individuals aged over 70, and non-shift workers; however, this correlation was not evident for all-cause, cancer, or other-cause mortality. Secondly, an overnight fasting duration of approximately 11.49 h correlated with the lowest risk for both all-cause and CVD mortality. Additionally, elderly individuals with the shortest fasting duration (≤7.5 h) exhibited elevated hazard ratios for both cancer and other-cause mortality. These findings underscore the complexity of fasting effects on health and could guide future research that may inform subsequent clinical guidelines.

To date, there has been limited research on the effects of TRE or IF within the elderly population [17,18,37]. Previous studies have investigated the effects of TRE on the elderly with varying sample sizes, ranging from 10 to 45 participants, whereas our study involved 10,561 participants. These studies also varied in observation durations, spanning from 4 to 12 weeks. Furthermore, they did not provide long-term observational results of TRE effects, whereas our study demonstrated a long-term follow-up time with a median of 6.66 years. Similarly, all the studies reported positive outcomes, including improvements in body composition, immune response, and walking speed. Only the study by Anton et al. identified minor side effects, such as headaches and dizziness, which were resolved with simple interventions [17]. In contrast, our study suggested that prolonged overnight fasting periods are associated with elevated CVD mortality in the elderly. Our findings introduce a new dimension to the discourse on TRE, highlighting the need for further exploration into the biological mechanisms that could explain the observed links between fasting duration and health outcomes.

Several factors may contribute to the negative effects of TRE on the elderly population. Firstly, extended fasting induces a metabolic shift from glycolysis to lipolysis and ketogenesis as the primary energy source [22]. This metabolic transition can lead to increased production of ketone bodies, which may induce oxidative stress and inflammation, potentially contributing to CVD risk [38]. Secondly, the fasting state can impair protein synthesis and promote muscle catabolism, as evidenced by reduced MyH4 expression, a key regulator of muscle structure and function [39]. This muscle degradation not only affects physical strength and mobility in the elderly but may also contribute to a negative nitrogen balance and compromised overall health. Moreover, the immune system is also adversely affected by prolonged fasting, with shifts in leukocyte migration potentially leading to a transiently weakened immune response [40]. Additionally, the stress response to fasting can lead to elevated cortisol levels [41], which may have deleterious effects on blood pressure regulation and cardiovascular health [42,43]. In light of these physiological impacts, our findings suggest that the benefits of TRE must be carefully weighed against potential risks, particularly in the elderly population.

In our study, a fasting duration of approximately 11 h corresponded to the lowest all-cause mortality rate among the elderly. The 11.49 h fasting mark may coincide with a metabolic sweet spot where the body has transitioned from relying primarily on glucose to utilizing fat stores effectively, thus avoiding the excessive catabolic state that could occur with prolonged fasting. After an initial period of glycogen depletion, the body enters a state of ketosis [22]; this metabolic shift could potentially explain the lower mortality risk observed at this fasting duration, as it allows efficient energy utilization without the detrimental effects of prolonged fasting. Additionally, the timing of this optimal fasting duration may also be significant in the context of circadian rhythms, which are integral to the regulation of metabolic processes [11]. A fasting period that aligns with the natural circadian cycle could enhance the body’s ability to restore and maintain metabolic homeostasis. Future research should aim to elucidate the biological processes that occur during this specific fasting window and how they influence health outcomes in the elderly population.

Our data indicated that the shortest length of fasting (≤7.5 h) was associated with an elevated hazard ratio for all-cause mortality (HR = 1.41) and other mortality (HR = 1.44), with *p* values just above the conventional threshold of 0.05. One possible explanation for this finding could be the presence of underlying health conditions that lead to both reduced fasting times and increased mortality risk. Individuals with compromised health may be unable to fast for extended periods due to medication schedules, nutritional needs, or other health-related factors [44]. Another consideration is the impact of social and behavioral factors on fasting duration. For some elderly individuals, the length of fasting periods may be a result of social engagement, cultural practices, or personal preferences that influence eating patterns [45]. The association between short fasting duration and higher mortality risk might also be indicative of disrupted circadian rhythms or sleep patterns [22], which are known to affect metabolic health and are common issues among older adults. The lack of statistical significance for the association between short fasting duration and mortality does not diminish the importance of these findings. The proximity to the threshold of 0.05 suggests that these associations might achieve significance with a larger sample size or a longer follow-up period [46]. The variability in the impact of fasting duration on different mortality types highlights the importance of considering a comprehensive range of health outcomes in future research.

By utilizing data from the NHANES, our study benefits from a selection process designed to be representative of the U.S. civilian population. Firstly, the NHANES employs a multistage, stratified sampling design that aims to capture a cross-section of the elderly population, taking into account factors such as geographic location, socioeconomic status, and ethnic diversity [24]. Secondly, we used survey weights in our analysis to account for the imbalances in the sample, further aligning the study cohort with the demographic characteristics of the U.S. elderly population [47]. Thirdly, the inclusion of participants with complete dietary and follow-up data helps ensure that the cohort reflects real-world dietary behaviors and health outcomes over an extended period, thus increasing the generalizability of the findings. Additionally, the study spans data from 2005 to 2018, covering multiple years and allowing a temporal representation of the elderly population’s dietary habits and health trends.

### Limitations

Several limitations need to be considered. Firstly, the presence of recall bias [48] and measurement error [48] in dietary self-reporting could affect the reliability of the fasting durations reported by the participants. To mitigate recall bias, we categorized participants based on their EI:EER ratio and adjusted for reporter status in our analysis. Additionally, we incorporated sensitivity analyses among the “acceptable reporters”, thereby enhancing the reliability of our findings [31]. Despite these efforts, we recognize that measurement error and social desirability bias were not explicitly addressed. Future research could benefit from incorporating objective measures of dietary intake, such as food diaries or digital tracking applications, to enhance the precision of dietary data collection. Secondly, while the NHANES survey is designed to capture a representative sample of dietary intake, including overnight fasting behaviors, the variability in the time gap might introduce variability that could affect the reported fasting durations. However, the study by Steinfeldt et al. [49] demonstrated that, on average, a time gap of 7 days between recalls in the NHANES survey did not compromise the consistency of dietary habit reporting. This finding indicates that participants can reliably report details that pertain to fasting duration, even with varying intervals between recalls. Thirdly, our analysis did not encompass comprehensive dietary information during non-fasting periods, such as the quality and composition of the diet or the eating schedules. This omission could potentially lead to an incomplete understanding of the overall dietary habits that may influence participant mortality. Moreover, the lack of detailed sleep period data and the absence of consecutive days of 24 h dietary recalls could introduce inaccuracies in estimating fasting periods. Sleep duration and quality are known to influence metabolic health and could interact with fasting duration in ways not captured by our study design. Furthermore, the dietary recall measurement of daily fasting length in the NHANES study did not provide information about the temporal relationship of cardiac risk factor development, diagnosis of cardiovascular disease or cancer, and when the daily fasting length began to exceed 12.38 h; thus, further research is needed on whether the longer fasting length preceded, was concurrent with, or occurred after the onset of risk factors and disease diagnoses. Given these considerations, our conclusions should be interpreted with an understanding of the study’s methodological boundaries. Future research, particularly randomized controlled trials that can account for these limitations, is needed to confirm our findings and provide a more definitive understanding of the impact of TRE on mortality.

## 5. Conclusions

Prolonged fasting periods are associated with a higher risk of cardiovascular mortality in the elderly population, although this correlation is not evident for all-cause, cancer, or other-cause mortality. A fasting duration of 11.49 h correlates with the lowest mortality risk. Additionally, elderly individuals with the shortest fasting duration exhibited elevated hazard ratios for both cancer and other-cause mortality. These findings should guide future research that may inform future clinical guidelines. As with any health intervention, clinicians should take caution to recommend a fasting regimen that is personalized to the health condition of people who are older. It is important to note that TRE as a health practice did not enter the public consciousness in the US until 2017–2018 or later; thus, few participants in this study would have deliberately used TRE, and further investigation of whether two 24 h recall measurements reflect the practice of long-term daily TRE is needed. Further research through randomized controlled trials should be conducted to comprehensively investigate the impact of TRE on mortality.

## Figures and Tables

**Table 1:** Baseline characteristics of study participants according to fasting duration categories.

Characteristics	Quartiles of Fasting Duration				
	Quartile 1 (≤7.5) *n* = 2710	Quartile 2 (>7.5 h, ≤10.58 h) *n* = 2575	Quartile 3 (>10.58 h, ≤12.38 h) *n* = 2655	Quartile 4 (>12.38 h) *n* = 2635	*p* Value
Age, mean (SE), years	69.53(0.20)	69.08(0.19)	69.64(0.20)	71.29(0.21)	<0.01
Age (categorial, 60–64,%)	29.00	32.22	29.75	23.33	<0.01
Gender (male, %)	40.93	49.05	47.20	45.14	0.03
Ethnicity (Non-Hispanic White, %)	83.87	82.60	77.97	69.33	<0.01
Educational level (some college or AA, %)	31.66	27.90	27.67	23.36	<0.01
Marital status (married, %)	61.41	62.44	67.52	57.26	<0.01
RIP (categorial, ≤1, %)	6.54	7.82	9.45	15.97	<0.01
Smoking (now, %)	11.26	13.37	8.11	10.35	0.01
Alcohol use (mild, %)	48.12	48.69	46.18	41.38	<0.01
BMI, (categorial, overweight, %), kg/m^2^	36.21	35.42	35.00	36.21	0.67
Diabetes (yes, %)	34.93	36.29	41.53	42.71	<0.01
CKD (yes, %)	31.91	30.26	32.09	41.02	<0.01
Depression (yes, %)	21.29	19.87	18.67	22.61	0.16
CVD (yes, %)	17.23	18.64	18.54	19.77	0.50
Hypertension (yes, %)	68.88	65.16	68.59	73.06	<0.01
Cancer (yes, %)	25.26	24.48	23.80	25.59	0.78
Fasting duration, mean (SE), hour	4.95 (0.06)	9.38 (0.02)	11.48 (0.02)	13.81 (0.03)	<0.01
DII, mean (SE)	1.26 (0.06)	1.42 (0.06)	1.41 (0.06)	1.97 (0.06)	<0.01
Deceased (%)	20.96	20.45	19.49	28.20	<0.01
Follow-up time, mean (SE), month	78.77 (1.84)	79.78 (1.85)	78.01 (1.70)	77.19 (1.83)	0.52
First meal (after 8 a.m., %)	80.04	75.27	50.15	24.72	<0.01
Late eating (after 9 p.m., %)	45.54	61.86	36.06	8.83	<0.01
EI/EER, mean (SE)	0.86 (0.01)	0.87 (0.01)	0.83 (0.01)	0.74 (0.01)	<0.01
Reporter status (acceptable, %)	30.93	36.25	33.57	27.38	<0.01
Shift work (yes, %)	1.20	1.87	0.66	0.73	<0.01
Sleep hour, mean (SE), hour	7.01 (0.04)	6.98 (0.04)	7.20 (0.04)	7.38 (0.05)	<0.01
Breakfast skipping (yes, %)	3.93	0.69	5.34	11.43	<0.01

Weighted percentage was used to represent categorical variables, while mean (standard error) was used to represent continuous variables. ANOVA for continuous variables and chi-square for categorical variables were used to calculate *p* value. Abbreviations: BMI, body mass index; CVD, cardiovascular disease; CKD, chronic kidney disease; DII, dietary inflammation index; EI, energy intake; EER, estimated energy requirement; RIP, ratio of income to poverty; SE, standard error; NHANES, National Health and Nutrition Examination Survey.

**Table 2:** Association between fasting duration, all-cause mortality, and cause-specific mortality according to fasting duration categories.

	Quartiles of Fasting Duration					
	Quartile 1	Quartile 2	Quartile 3	Quartile 4	*p* Value ^e^	Per 1 h Increment in Fasting Duration *
All-cause mortality ^c^	1.12 (0.93, 1.34)	1.00 (0.84, 1.19)	1.00 (Ref)	1.10 (0.89, 1.36)	0.55	1.00 (0.98, 1.02)
CVD mortality ^c^	0.95 (0.70, 1.30)	0.84 (0.62, 1.14)	1.00 (Ref)	**1.58 (1.10, 2.28)**	**0.04**	**1.04 (1.00, 1.09)**
Cancer mortality ^d^	1.41 (0.99, 2.01)	1.35 (0.95, 1.91)	1.00 (Ref)	0.87 (0.62, 1.23)	0.43	**0.95 (0.91, 0.98)**
Other mortality ^b^	1.44 (0.98, 2.13)	1.23 (0.91, 1.65)	1.00 (Ref)	1.21 (0.89, 1.62)	0.22	0.98 (0.95, 1.02)

Results were presented as hazard ratios (95% confidence intervals). Significant values are in bold (*p* < 0.05). ^b^ Other mortality was adjusted for age (categorial), gender, educational level, marital status, ratio of income to poverty (categorial), ethnicity, diabetes, chronic kidney disease, body mass index, depression, smoking, alcohol use, dietary inflammatory index, shift work, first meal, late eating, sleep hour, reporter status, and breakfast skipping. ^c^ CVD mortality and all-cause mortality were further adjusted (from other mortality) for hypertension and CVD. ^d^ Cancer mortality was further adjusted (from other mortality) for cancer. Quartile ranges: Quartile 1: ≤7.5 h; Quartile 2: >7.5 h, ≤10.58 h; Quartile 3: >10.58 h, ≤12.38 h; Quartile 4: >12.38 h. ^e^ *p* value refers to FDR-corrected *p* value of Quartile 4. * Hazard ratios for each hour increment in fasting duration. Abbreviation: CVD, cardiovascular disease; Ref, reference.

## Data Availability

Publicly available datasets were analyzed in this study. All the raw data used in this study are derived from the public NHANES data portal (https://wwwn.cdc.gov/nchs/nhanes/analyticguidelines.aspx, accessed on 25 March 2024).

## Supplementary Materials

The following supporting information can be downloaded at: https://www.mdpi.com/article/10.3390/nu16132018/s1, Figure S1. Histogram for demonstration of distribution of fasting duration among elderly in NHANES 2005-2018. Figure S2. Non-linear associations between fasting duration and cancer-specific (A), and other-cause (B) mortality. Figure S3. Non-linear association between fasting duration and all-cause mortality among population aged 18–59 from NHANES 2005–2018. Table S1. The comparison between 2 days dietary recalls among the elderly population (*n* = 10,561) in NHANES 2005–2018. Table S2. The fasting duration distribution of Day 1 dietary recall and Day 2 dietary recalls. Table S3. Detailed characteristics of study participants according to fasting duration categories. Table S4. Association between fasting duration, all-cause mortality, and cause-specific mortality according to fasting duration categories among Non-Hipanic white (*n* = 5493). Table S5. Association between fasting duration, all-cause mortality, and cause-specific mortality according to fasting duration using 12.38 h as cut-off value and population after matching. Table S6. Association between fasting duration, all-cause mortality, and cause-specific mortality according to fasting duration categories. Table S7. Detailed characteristics of study participants (recoded educational level, and RIP) according to fasting duration categories. Table S8. Detailed characteristics of study participants according to fasting duration categories after matching.
