# Supplementary material for: A National Study Exploring the Association between Fasting Duration and Mortality among the Elderly
Source: Nutrients. 2024 Jun 26;16(13):2018. doi: 10.3390/nu16132018 (PMC11242983; doi:10.3390/nu16132018)
Supplement: Supplementary file 1 [file nutrients-16-02018-s001.zip › nutrients-2974643-supplementary.pdf]

Supplemental Materials

A National Study Exploring the Association Between Fasting Duration and Mortality Among the Elderly

Figure S1. Histogram for demonstration of distribution of fasting duration among elderly in NHANES 2005-2018. 2
Figure S2. Non-linear associations between fasting duration and cancer-specific (A) and other-cause (B) mortality. 3
Figure S3. Non-linear association between fasting duration and all-cause mortality among population aged 18-59 from NHANES 2005-2018. 4
Table S1. The comparison between 2-day dietary recalls among the elderly population (n=10,561) in NHANES 2005-2018. 5
Table S2. The fasting duration distribution of day 1 dietary recall and day 2 dietary recall. 5
Table S3. Detailed characteristics of study participants according to fasting duration categories. 6
Table S4. Association between fasting duration, all-cause mortality, and cause-specific mortality according to fasting duration categories among Non-Hispanic White participants (n=5,493). 10
Table S5. Association between fasting duration, all-cause mortality, and cause-specific mortality according to fasting duration using 12.38 hours as cut-off value and population after matching. 11
Table S6. Association between fasting duration, all-cause mortality, and cause-specific mortality according to fasting duration categories 12
Table S7. Detailed characteristics of study participants (educational level and RIP) according to fasting duration categories. 13
Table S8. Detailed characteristics of study participants according to fasting duration categories after matching. 14

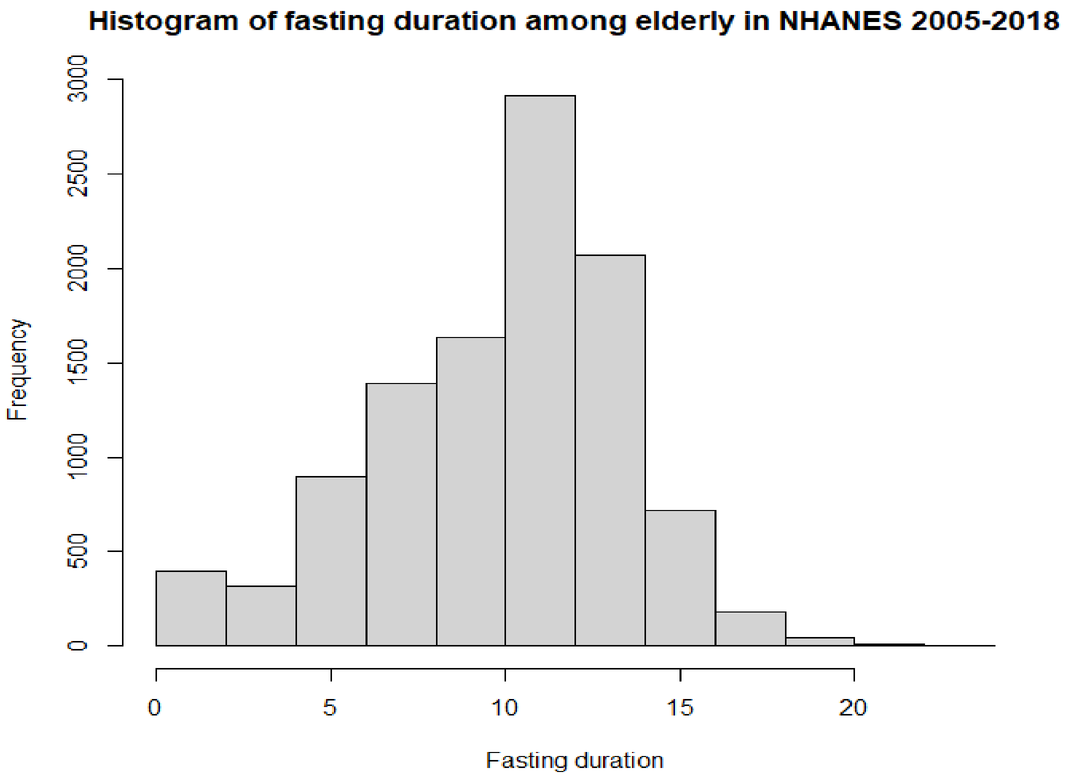

Figure S1. Histogram for demonstration of distribution of fasting duration among elderly in NHANES 2005-2018.

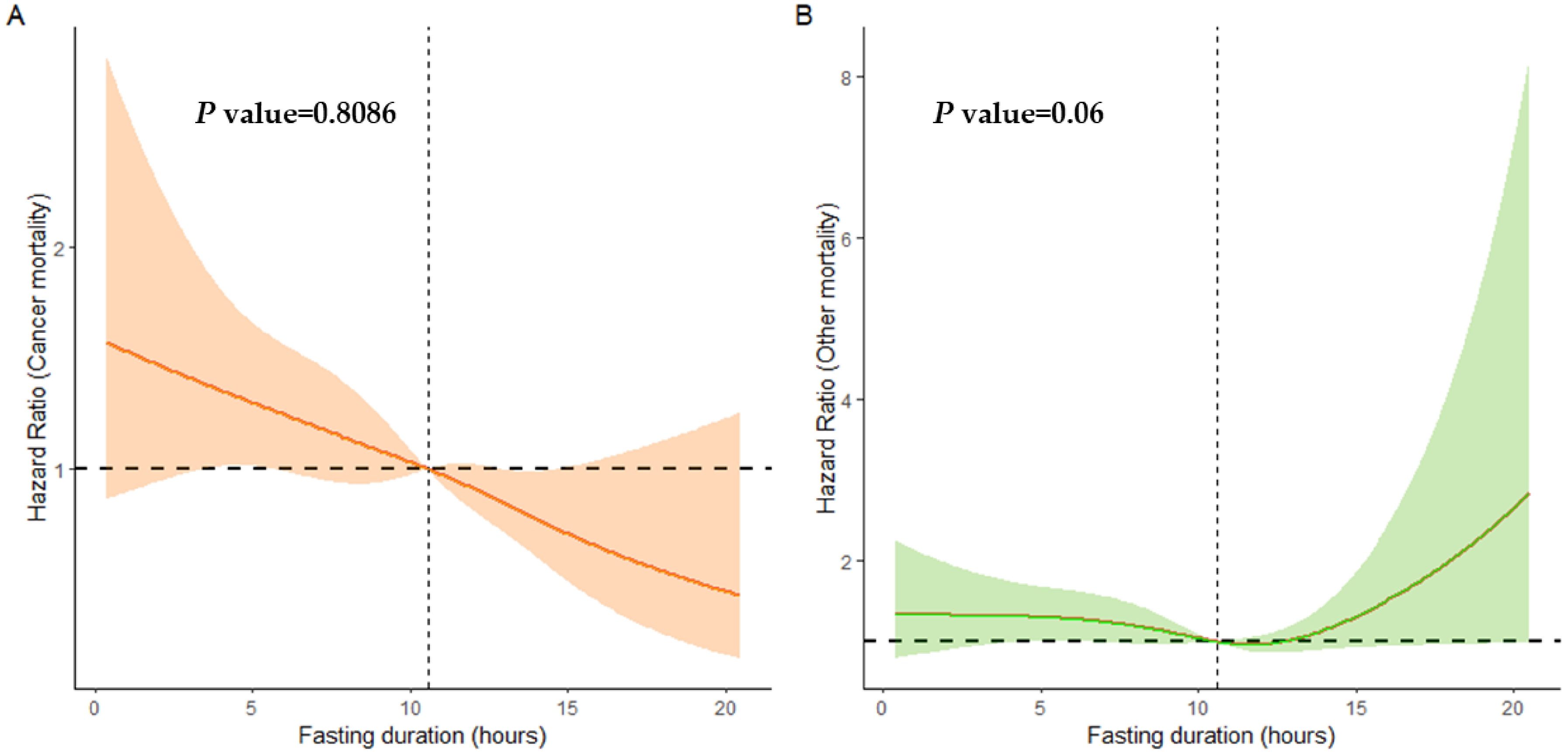

Figure S2. Non-linear associations between fasting duration and cancer-specific (A) and other-cause (B) mortality.

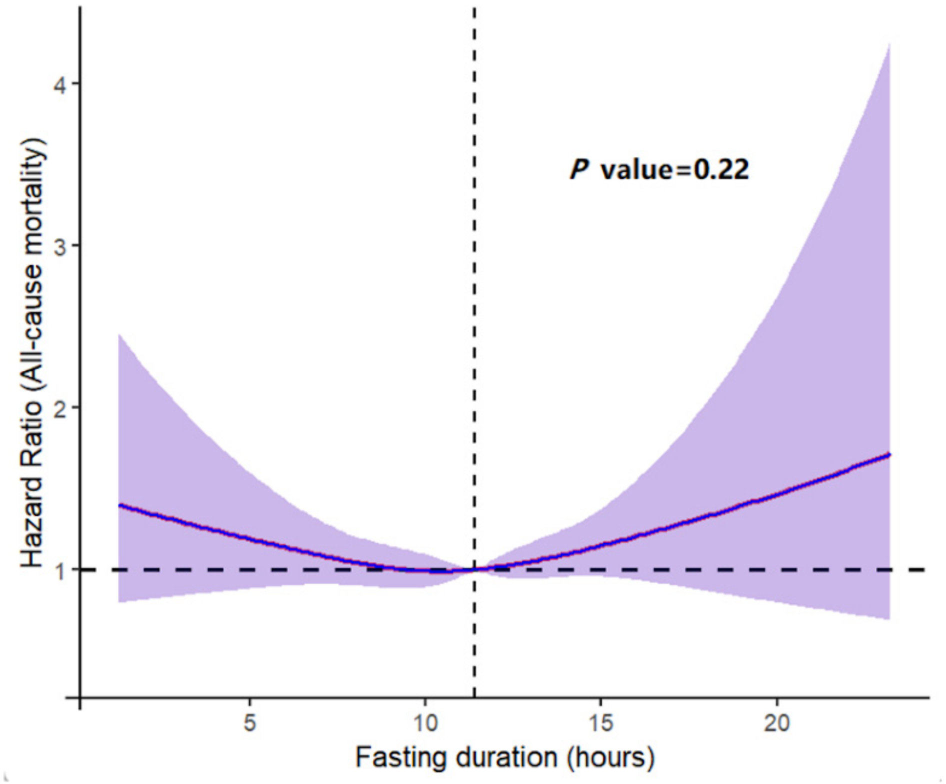

Figure S3. Non-linear association between fasting duration and all-cause mortality among population aged 18-59 from NHANES 2005-2018.

Solid purple lines represent hazard ratios adjusted for age (Categorial), gender, educational level, marital status, ratio of income to poverty (Categorial), ethnicity, diabetes, chronic kidney disease, body mass index, depression, smoking, use of alcohol, dietary inflammatory index, hypertension, and cardiovascular disease, with light areas showing the 95% confidence intervals derived from restricted cubic spline regressions.

Abbreviation: BMI, Body Mass Index; CVD, Cardiovascular Disease; CKD, Chronic Kidney Disease; DII, Dietary Inflammatory Index

Table S1. The comparison between 2-day dietary recalls among the elderly population (n=10,561) in NHANES 2005-2018.

	**Day 1**	**Day 2**	***P* value**
Time of first calories intake (hours, mean, SE)	7.42 (0.04)	7.35 (0.04)	0.06
Time of last calories intake (hours, mean, SE)	20.33 (0.03)	20.41 (0.03)	**<0.01**
Fasting duration (hours, mean, SE)	9.48 (0.07)	9.44 (0.06)	0.63
Energy intake (kcal, mean, SE)	1871.55 (13.66)	1845.92 (13.71)	0.07

Weighted mean (standard error) was used to represent continuous variables. ANOVA for continuous variables were used to calculate *P* value. Abbreviations: NHANES, National Health, and Nutrition Examination Survey.

Table S2. The fasting duration distribution of day 1 dietary recall and day 2 dietary recall.

	**Mean**	**SD**	**N**	**Min**	**25th percentile**	**50th percentile**	**75th percentile**	**Max**	**IQR**
Day 1	10.04	4.51	11806	0.08	8.5	11	13	24	4.5
Day 2	9.95	4.44	10575	0.08	8.5	11	12.75	24	4.25

Results are presented as hours.

Abbreviation: SD, standard deviation; N, number; IQR, interquartile range.

Table S3. Detailed characteristics of study participants according to fasting duration categories.

Characteristics	Quartiles of fasting duration				*P* value
	Quartile 1 (≤7.5) n=2,710	Quartile 2 (>7.5h, ≤10.58h) n=2,575	Quartile 3 (>10.58h, ≤12.38h) n=2,655	Quartile 4 (>12.38h) n=2,635	
Age, mean (SE), years	69.53 (0.20)	69.08 (0.19)	69.64 (0.20)	71.29 (0.21)	<0.01
Age (Categorical, %)					< 0.01
60-64	29.00	32.22	29.75	23.33	
65-69	25.92	25.27	22.48	20.60	
70-74	18.59	17.06	19.83	19.03	
≥75	26.49	25.45	27.93	37.04	
Gender (%)					0.03
Male	40.93	49.05	47.20	45.14	
Female	59.07	50.95	52.80	54.86	
Ethnicity (%)					< 0.01
Mexican American	2.64	2.91	4.67	7.48	
Other Hispanic	1.76	3.29	4.61	4.10	
Non-Hispanic white	83.87	82.60	77.97	69.33	
Non-Hispanic black	6.24	7.53	7.69	14.49	
Other Race(s)	5.48	3.66	5.07	4.60	
Educational level (%)					< 0.01
Less than 9th grade	5.01	5.51	7.88	13.29	
9-11th grade	9.00	10.67	7.94	14.76	
High school graduate	23.51	24.86	25.53	27.58	
Some college or AA	31.66	27.90	27.67	23.36	
College or above	30.83	31.06	30.99	21.00	
Marital status (%)					< 0.01
Married	61.41	62.44	67.52	57.26	
Widowed	19.00	18.13	17.00	21.25	
Divorced	13.35	12.89	9.49	11.87	
Separated	0.97	1.70	0.94	1.85	

Never married	2.87	3.19	3.65	4.97	
Living with a partner	2.40	1.64	1.40	2.80	
RIP (Categorial, %)					< 0.01
≤1	6.54	7.82	9.45	15.97	
＞1, ≤3	39.66	38.97	39.77	46.71	
＞3	53.80	53.20	50.78	37.32	
Smoking (%)					0.01
Never	48.06	48.46	49.20	51.42	
Former	40.68	38.17	42.69	38.23	
Now	11.26	13.37	8.11	10.35	
Alcohol user (%)					< 0.01
Mild	48.12	48.69	46.18	41.38	
Moderate	12.36	11.40	12.54	9.06	
Former	22.57	19.51	20.72	24.05	
Heavy	5.72	5.66	6.62	7.90	
Never	11.23	14.75	13.93	17.61	
First meal (%)					< 0.01
Before 8 a.m.	19.96	24.73	49.85	75.28	
After 8 a.m.	80.04	75.27	50.15	24.72	
Late eating (%)					<0.01
Before 9 p.m.	54.46	38.14	63.94	91.17	
After 9 p.m.	45.54	61.86	36.06	8.83	
BMI, (Categorial, %), kg/m^2					0.67
Normal (<25)	25.02	26.19	23.19	24.77	
Overweight (≥25, <30)	36.21	35.42	35.00	36.21	
Obese (≥30)	38.78	38.40	41.81	39.03	
Diabetes (%)					< 0.01
No	65.07	63.71	58.47	57.29	
Yes	34.93	36.29	41.53	42.71	
CKD (%)					< 0.01

No	68.09	69.74	67.91	58.98	
Yes	31.91	30.26	32.09	41.02	
Depression (%)					0.16
No	78.71	80.13	81.33	77.39	
Yes	21.29	19.87	18.67	22.61	
CVD (%)					0.5
No	82.77	81.36	81.46	80.23	
Yes	17.23	18.64	18.54	19.77	
Hypertension (%)					< 0.01
No	31.12	34.84	31.41	26.94	
Yes	64.82	65.21	70.05	72.98	
Cancer (%)					0.78
No	74.74	75.52	76.20	74.41	
Yes	68.88	65.16	68.59	73.06	
Fasting duration, mean (SE), hour	4.95 (0.06)	9.38 (0.02)	11.48 (0.02)	13.81 (0.03)	< 0.01
DII, mean (SE)	1.26 (0.06)	1.42 (0.06)	1.41 (0.06)	1.97 (0.06)	< 0.01
Living status (%)					< 0.01
Alive	79.04	79.55	80.51	71.80	
Deceased	20.96	20.45	19.49	28.20	
Follow-up time, mean (SE), month	78.77 (1.84)	79.78 (1.85)	78.01 (1.70)	77.19 (1.83)	0.52
Sleeping hour, mean (SE), hour	7.01 (0.04)	6.98 (0.04)	7.20 (0.04)	7.38 (0.05)	<0.01
EI/EER, mean (SE)	0.86 (0.01)	0.87 (0.01)	0.83 (0.01)	0.74 (0.01)	<0.01
Reporter status (%)					<0.01
Under-reporter	8.82	10.89	12.09	16.05	
Acceptable reporter	30.93	36.25	33.57	27.38	
Over-reporter	0.50	1.33	0.85	0.18	
Missing	59.76	51.54	53.49	56.38	
Shift worker (%)					<0.01
No	7.96	9.29	7.73	5.79	
Yes	1.20	1.87	0.66	0.73	

Missing	90.84	88.84	91.61	93.48	
Breakfast skipping (Yes, %)					<0.01
No	96.07	99.31	94.66	88.57	
Yes	3.93	0.69	5.34	11.43	

Abbreviations: BMI, Body Mass Index; CVD, Cardiovascular Disease; CKD, Chronic Kidney Disease; DII, Dietary Inflammation Index; EI, Energy Intake; EER, Estimated Energy Requirement; RIP, Ratio of Income to Poverty; SE, Standard Error; NHANES, National Health, and Nutrition Examination Survey.

Table S4. Association between fasting duration, all-cause mortality, and cause-specific mortality according to fasting duration categories among Non-Hispanic White participants (n=5,493).

	Quartiles of fasting duration					
	Quartile 1	Quartile 2	Quartile 3	Quartile 4	*P* value [e]	Per 1 h increment in fasting duration *
All-cause mortality [c]	1.11 (0.92,1.36)	0.97 (0.80,1.18)	1.00 (Ref)	1.08 (0.84,1.39)	0.78	1.00 (0.97,1.02)
CVD mortality [c]	0.90 (0.64,1.26)	0.71 (0.48,1.04)	1.00 (Ref)	**1.85 (1.25,2.76)**	**0.006**	**1.06 (1,00,1.12)**
Cancer mortality [d]	1.47 (0.96,2.26)	1.48 (1.02,2.16)	1.00 (Ref)	0.72 (0.46,1.13)	0.16	0.93 (0.89,0.97)
Other mortality [b]	1.51 (0.95,2.41)	1.25 (0.88,1.77)	1.00 (Ref)	1.17 (0.79,1.72)	0.43	0.97 (0.93,1.01)

Results were presented as hazard ratios (95% confidence intervals). Significant values in bold (*P*< 0.05).

[b] Other mortality was adjusted for age (Categorial), gender, educational level, marital status, ratio of income to poverty (Categorial), diabetes, chronic kidney diseases, body mass index, depression, smoking, alcohol user, dietary inflammatory index, shift worker, first meal, late eating, sleep hour, reporter status and breakfast skipping.

[c] CVD mortality and all-cause mortality were further adjusted (from other mortality) for hypertension and CVD.

[d] Cancer mortality was further adjusted (from other mortality) for cancer. Quartile ranges: Quartile 1:≤7.5h; Quartile 2:>7.5h, ≤10.58h; Quartile 3:>10.58h,≤12.38h; Quartile 4:>12.38h.

[e] P value refers to FDR-corrected P value of Quartile 4.

*Hazard ratios for each hour increment in fasting duration.

Abbreviation: CVD, Cardiovascular Disease; Ref, reference

Table S5. Association between fasting duration, all-cause mortality, and cause-specific mortality according to fasting duration using 12.38 hours as cut-off value and population after matching.

	**Fasting duration(≤12.38h) n=2,618**	**Fasting duration(>12.38h) n=1,808**	***P* value**
All-cause mortality [c]	1.00 (Ref)	1.06(0.92,1.22)	0.40
CVD mortality [c]	1.00 (Ref)	1.55(1.02, 2.35)	0.04
Cancer mortality [d]	1.00 (Ref)	0.71(0.51, 1.00)	0.05
Other mortality [b]	1.00 (Ref)	1.39(0.98, 1.97)	0.06

Results were presented as hazard ratios (95% confidence intervals). Significant values in bold ($P< 0.05$).

[b] Other mortality was adjusted for age, gender, educational level, marital status, ratio of income to poverty (Categorial), diabetes, chronic kidney diseases, body mass index, depression, smoking, alcohol user, dietary inflammatory index, shift worker, first meal, late eating, sleeping hour, reporter status and breakfast skipping.

[c] CVD mortality and all-cause mortality were further adjusted (from other mortality) for hypertension and CVD.

[d] Cancer mortality was further adjusted (from other mortality) for cancer.

Abbreviation: CVD, Cardiovascular Disease; Ref, reference

Table S6. Association between fasting duration, all-cause mortality, and cause-specific mortality according to fasting duration categories.

	Quartiles of fasting duration					
	**Quartile 1**	**Quartile 2**	**Quartile 3**	**Quartile 4**	***P* value [e]**	**Per 1 h increment in fasting duration ***
All-cause mortality [c]	1.12 (0.93,1.34)	1.00 (0.84,1.20)	1.00 (Ref)	1.10 (0.89,1.36)	0.60	1.00 (0.98,1.02)
CVD mortality [c]	0.96 (0.70,1.31)	0.83 (0.61,1.12)	1.00 (Ref)	**1.56 (1.07,2.27)**	**0.05**	**1.04 (1,00,1.09)**
Cancer mortality [d]	1.38 (0.98,1.94)	1.29 (0.92,1.82)	1.00 (Ref)	0.90 (0.65,1.24)	0.52	0.95 (0.92,0.99)
Other mortality [b]	1.41 (0.94,2.11)	1.24 (0.93,2.11)	1.00 (Ref)	1.28 (0.94,1.73)	0.15	0.99 (0.96,1.03)

Results were presented as hazard ratios (95% confidence intervals). Significant values in bold ($P< 0.05$).

[b] Other mortality was adjusted for age (Categorial), ethnicity, gender, educational level (Recode), marital status, ratio of income to poverty (Categorial, Recode), diabetes, chronic kidney diseases, body mass index, depression, smoking, alcohol user, dietary inflammatory index, shift worker, first meal, late eating, sleep hour, reporter status and breakfast skipping.

[c] CVD mortality and all-cause mortality were further adjusted (from other mortality) for hypertension and CVD.

[d] Cancer mortality was further adjusted (from other mortality) for cancer. Quartile ranges: Quartile 1:≤7.5h; Quartile 2:>7.5h, ≤10.58h; Quartile 3:>10.58h,≤12.38h; Quartile 4:>12.38h.

[e] P value refers to FDR-corrected P value of Quartile 4.

*Hazard ratios for each hour increment in fasting duration.

Abbreviation: CVD, Cardiovascular Disease; Ref, reference

Table S7. Detailed characteristics of study participants (educational level and RIP) according to fasting duration categories.

Characteristics	Quartiles of fasting duration				
	**Quartile 1 (≤7.5h) n=2,710**	**Quartile 2 (>7.5h, ≤10.58h) n=2,575**	**Quartile 3 (>10.58h, ≤12.38h) n=2,655**	**Quartile 4 (>12.38h) n=2,635**	***P* value**
Educational level (Recode, %)					< 0.01
Never attended high school	14.01	16.17	15.82	28.06	
High school or above	85.99	83.83	84.18	71.94	
RIP (Categorial, Recode, %)					< 0.01
≤3	46.20	46.80	49.22	62.68	
> 3	53.80	53.20	50.78	37.32	

Weighted percentage was used to represent categorical variables, while mean (standard error) was used to represent continuous variables. ANOVA for continuous variables and Chi-Square for categorical variables were used to calculate *P* value.

Abbreviations: BMI, Body Mass Index; CVD, Cardiovascular Disease; CKD, Chronic Kidney Disease; DII, Dietary Inflammation Index; EI, Energy Intake; EER, Estimated Energy Requirement; RIP, Ratio of Income to Poverty; SE, Standard Error; NHANES, National Health, and Nutrition Examination Survey.

Table S8. Detailed characteristics of study participants according to fasting duration categories after matching.

Characteristics	Fasting duration (≤12.38h) n=2,608	Fasting duration (>12.38h) n=1,808	*P* value
Sleeping hour (Categorical)			0.42
≥7 hours	987 (37.7)	703 (38.9)	
<7 hours	823 (31.4)	580 (32.1)	
Missing	808 (30.9)	525 (29.0)	
Follow-up time (months)	78.58 (45.98)	78.02 (47.42)	0.70
Deceased			0.07
No	1901 (72.6)	1267 (70.1)	
Yes	717 (27.4)	541 (29.9)	
Deceased (CVD causes)			1.00
No	2429 (92.8)	1677 (92.8)	
Yes	189 (7.2)	131 (7.2)	
Deceased (Other causes)			0.45
No	2405 (91.9)	1673 (92.5)	
Yes	213 (8.1)	135 (7.5)	
Deceased (Cancer causes)			0.02
No	2468 (94.3)	1673 (92.5)	
Yes	150 (5.7)	135 (7.5)	
Age	70.67 (6.99)	70.80 (7.12)	0.53
DII	1.85 (1.79)	1.91 (1.82)	0.26
Gender			0.93
Male	1294 (49.4)	897 (49.6)	
Female	1324 (50.6)	911 (50.4)	
Cancer			0.90
No	2071 (79.1)	1434 (79.3)	
Yes	547 (20.9)	374 (20.7)	
Alcohol user			0.99
Mild	837 (32.0)	586 (32.4)	

Moderate	235 (9.0)	167 (9.2)	
Former	639 (24.4)	433 (23.9)	
Heavy	200 (7.6)	138 (7.6)	
Never	467 (17.8)	312 (17.3)	
Missing	240 (9.2)	172 (9.5)	
Marital status			0.93
Married	1407 (53.7)	957 (52.9)	
Widowed	596 (22.8)	419 (23.2)	
Divorced	327 (12.5)	223 (12.3)	
Separated	86 (3.3)	57 (3.2)	
Never married	133 (5.1)	105 (5.8)	
Living with a partner	69 (2.6)	47 (2.6)	
Educational level			0.79
Less than 9th grade	486 (18.6)	321 (17.8)	
9-11th grade	412 (15.7)	297 (16.4)	
High school graduate	654 (25.0)	472 (26.1)	
Some college or AA	606 (23.1)	416 (23.0)	
College or above	460 (17.6)	302 (16.7)	
RIP (Categorical)			0.72
≤1	466 (17.8)	333 (18.4)	
>1, ≤3	1171 (44.7)	828 (45.8)	
>3	707 (27.0)	467 (25.8)	
Missing	274 (10.5)	180 (10.0)	
Depression			0.86
No	1895 (72.4)	1298 (71.8)	
Yes	574 (21.9)	401 (22.2)	
Missing	149 (5.7)	109 (6.0)	
Smoking			0.62
Never	1344 (51.3)	903 (49.9)	
Former	970 (37.1)	683 (37.8)	

Now	304 (11.6)	222 (12.3)	
BMI (Categorial)			0.91
Normal (<25)	616 (23.5)	442 (24.4)	
Overweight (≥25, <30)	939 (35.9)	638 (35.3)	
Obese (≥30)	1004 (38.3)	686 (37.9)	
Missing	59 (2.3)	42 (2.3)	
CVD			0.67
No	2097 (80.1)	1438 (79.5)	
Yes	521 (19.9)	370 (20.5)	
Hypertension			0.55
No	722 (27.6)	483 (26.7)	
Yes	1896 (72.4)	1325 (73.3)	
Ethnicity			0.56
Mexican American	367 (14.0)	243 (13.4)	
Other Hispanic	250 (9.5)	162 (9.0)	
Non-Hispanic white	1228 (46.9)	839 (46.4)	
Non-Hispanic black	620 (23.7)	466 (25.8)	
Other Race(s)	153 (5.8)	98 (5.4)	
Diabetes			0.79
Yes	1221 (46.6)	835 (46.2)	
No	1397 (53.4)	973 (53.8)	
Reporter status			0.31
Under-reporter	468 (17.9)	345 (19.1)	
Acceptable reporter	775 (29.6)	520 (28.8)	
Over-reporter	20 (0.8)	7 (0.4)	
Missing	1355 (51.8)	936 (51.8)	
Shift worker			0.54
No	167 (6.4)	120 (6.6)	
Yes	32 (1.2)	16 (0.9)	
Missing	2419 (92.4)	1672 (92.5)	

Late eating			<0.01
Before 8 a.m.	2015 (77.0)	1496 (82.7)	
After 8 a.m.	603 (23.0)	312 (17.3)	
First meal			<0.01
Before 8 a.m.	1530 (58.4)	1263 (69.9)	
After 8 a.m.	1088 (41.6)	545 (30.1)	
CKD			0.01
No	1521 (61.7)	989 (57.8)	
Yes	946 (38.3)	721 (42.2)	
Fasting duration (hours)	9.28 (2.96)	13.82 (1.36)	<0.01

Mean (SD) was used to represent continuous variables. Number of participants(percentage) was used to present categorical variables. ANOVA for continuous variables and Chi-square for categorical variables were used to calculate *P* value.

Abbreviations: BMI, Body Mass Index; CVD, Cardiovascular Disease; CKD, Chronic Kidney Disease; DII, Dietary Inflammation Index; RIP, Ratio of Income to Poverty; SD, Standard Deviation; NHANES, National Health, and Nutrition Examination Survey
